# Multiple policies to enhance prescribing efficiency for established medicines in Europe with a particular focus on demand-side measures: findings and future implications

**DOI:** 10.3389/fphar.2014.00106

**Published:** 2014-06-17

**Authors:** Brian Godman, Bjorn Wettermark, Menno van Woerkom, Jessica Fraeyman, Samantha Alvarez-Madrazo, Christian Berg, Iain Bishop, Anna Bucsics, Stephen Campbell, Alexander E. Finlayson, Jurij Fürst, Kristina Garuoliene, Harald Herholz, Marija Kalaba, Ott Laius, Jutta Piessnegger, Catherine Sermet, Ulrich Schwabe, Vera V. Vlahović-Palčevski, Vanda Markovic-Pekovic, Luka Vončina, Kamila Malinowska, Corinne Zara, Lars L. Gustafsson

**Affiliations:** ^1^Division of Clinical Pharmacology, Department of Laboratory Medicine, Karolinska Institutet, Karolinska University Hospital HuddingeStockholm, Sweden; ^2^Medicines Use and Health, Strathclyde Institute of Pharmacy and Biomedical Sciences, University of StrathclydeGlasgow, UK; ^3^Liverpool Health Economics Centre, University of Liverpool Management SchoolLiverpool, UK; ^4^Centre for Pharmacoepidemiology, Karolinska Institute, Karolinska University HospitalSolna, Stockholm, Sweden; ^5^Department of Healthcare Development, Public Healthcare Services Committee, Stockholm County CouncilStockholm, Sweden; ^6^Dutch Institute for Rational Use of MedicinesUtrecht, Netherlands; ^7^Epidemiology and Social Medicine, Research Group Medical Sociology and Health Policy, University of AntwerpAntwerp, Belgium; ^8^Division of Epidemiology, Department of Pharmacoepidemiology, Norwegian Institute of Public HealthOslo, Norway; ^9^Public Health and Intelligence Business Unit, National Services NHS ScotlandEdinburgh, UK; ^10^Department of Finance, Faculty of Business, Economics and Statistics, University of ViennaVienna, Austria; ^11^Department of Reimbursement, Hauptverband der Österreichischen SozialversicherungsträgerVienna, Austria; ^12^Centre for Primary Care, Institute of Population Health, University of ManchesterManchester, UK; ^13^Green Templeton College, University of OxfordOxford, UK; ^14^Health Insurance InstituteLjubljana, Slovenia; ^15^Department of Pathology, Forensic Medicine and Pharmacology, Faculty of Medicine, University of VilniusVilnius, Lithuania; ^16^Medicines Reimbursement Department, National Health Insurance FundVilnius, Lithuania; ^17^Kasemarzliche Vereinigung HessenFrankfurt am Main, Germany; ^18^Department of Medicines and Pharmacoeconomics, Republic Fund for Health InsuranceBelgrade, Serbia; ^19^State Agency of MedicinesTartu, Estonia; ^20^IRDESParis, France; ^21^Institute of Pharmacology, University of HeidelbergHeidelberg, Germany; ^22^Department of Clinical Pharmacology, University Hospital RijekaRijeka, Croatia; ^23^Faculty of Medicine, University of Banja LukaBanja Luka, Republic Srpska, Bosnia and Herzegovina; ^24^Ministry of Health and Social WelfareBanja Luka, Republic Srpska, Bosnia and Herzegovina; ^25^Ministry of HealthRepublic of Croatia, Zagreb, Croatia; ^26^Department of Epidemiology and Health Promotion, Public Health SchoolWarsaw, Poland; ^27^Drug Management Department, National Health FundWarsaw, Poland; ^28^Barcelona Health Region, Catalan Health ServiceBarcelona, Spain

**Keywords:** demand-side measures, drug utilization studies, generics, PPIs, renin-angiotensin inhibitor drugs, statins

## Abstract

**Introduction**: The appreciable growth in pharmaceutical expenditure has resulted in multiple initiatives across Europe to lower generic prices and enhance their utilization. However, considerable variation in their use and prices.

**Objective**: Assess the influence of multiple supply and demand-side initiatives across Europe for established medicines to enhance prescribing efficiency before a decision to prescribe a particular medicine. Subsequently utilize the findings to suggest potential future initiatives that countries could consider.

**Method**: An analysis of different methodologies involving cross national and single country retrospective observational studies on reimbursed use and expenditure of PPIs, statins, and renin-angiotensin inhibitor drugs among European countries.

**Results**: Nature and intensity of the various initiatives appreciably influenced prescribing behavior and expenditure, e.g., multiple measures resulted in reimbursed expenditure for PPIs in Scotland in 2010 56% below 2001 levels despite a 3-fold increase in utilization and in the Netherlands, PPI expenditure fell by 58% in 2010 vs. 2000 despite a 3-fold increase in utilization. A similar picture was seen with prescribing restrictions, i.e., (i) more aggressive follow-up of prescribing restrictions for patented statins and ARBs resulted in a greater reduction in the utilization of patented statins in Austria vs. Norway and lower utilization of patented ARBs vs. generic ACEIs in Croatia than Austria. However, limited impact of restrictions on esomeprazole in Norway with the first prescription or recommendation in hospital where restrictions do not apply. Similar findings when generic losartan became available in Western Europe.

**Conclusions**: Multiple demand-side measures are needed to influence prescribing patterns. When combined with supply-side measures, activities can realize appreciable savings. Health authorities cannot rely on a “spill over” effect between classes to affect changes in prescribing.

## Introduction

**Pharmaceutical expenditure** is under increasing scrutiny, with expenditure rising by more than 50% in real terms during the past decade among OECD countries (Godman et al., 2013a). As a result, pharmaceutical expenditure has become the largest, or equaling the largest, cost component in ambulatory care and in some countries up to 60% of total healthcare expenditure (Godman et al., 2012a, 2013a). This will continue unless actively addressed, driven by well-known factors including ageing populations, rising patient expectations and the continued launch of new premium priced technologies (Garattini et al., 2008; Godman et al., 2013a).

KEY CONCEPT 1. Pharmaceutical expenditure Pharmaceutical expenditure is under increasing scrutiny among health authorities, which is already resulting in some countries unable to fund new premium priced drugs. Potential ways forward include demand-side measures to encourage the preferential prescribing of low cost generics where care is not compromised, including addressing concerns with generics where these exist.

We are already seeing some countries unable to fund new premium priced drugs due to continued pressures. If not addressed, the number of countries is likely to grow with prices of new drugs, including new biological drugs, typically between US$100,000–US$400,000 per patient per year or more (Experts in chronic myeloid leukemia, 2013; Godman et al., 2013a; Malmström et al., 2013). Measures for new drugs include instigating models to optimize their managed entry, starting pre-launch and continuing post-launch (Malmström et al., 2013). They also include the development of managed entry agreements to enhance the value of new drugs, and hence potential funding, as well as registries post-launch to assess the effectiveness and safety of new drugs in routine clinical care (Klemp et al., 2011; Ferrario and Kanavos, 2013; Malmström et al., 2013). Greater discussion of these activities including their rationale are outside the scope of this article.

Measures for established drugs include initiatives to increase the prescribing of low cost generic drugs vs. originators and patented products in a class where all products in the class or related class are seen as essentially therapeutically similar in all or nearly all patients. As a result, considerable savings can be achieved without compromising care (Bennie et al., 2012; Godman et al., 2012a, 2013a). The savings are facilitated by annual global sales of pharmaceutical products losing their patents between 2008 and 2013 estimated at US$50 to 100 billion (€35–70). This rises to US$255 billion between 2011 and 2016 (Godman et al., 2012a, 2013b). There are also central procurement initiatives among countries to conserve resources. These include a public tendering system for simvastatin in Belgium, two-weekly assessments of prices for multiple sourced products in Denmark, monthly auctions for generics in Sweden and up to yearly tenders in the Netherlands (Dylst et al., 2011, 2013; Fraeyman et al., 2013; Godman et al., 2013a).

Demand-side measures to enhance the prescribing of generics vs. originators and patented products in a class include educational activities, prescribing targets, financial incentives including patient co-payment differentials, compulsory International Non-proprietary Name (INN) prescribing and prescribing restrictions (Godman et al., 2012a, 2013a). This includes promoting generics even when they are available initially as different salts to the originator with a lower number of indications once bioequivalence has been demonstrated, e.g., generic clopidogrel (Baumgärtel et al., 2012). In addition, setting minimum quotas (in percentages) for physicians to encourage them to prescribe low-cost medicines, e.g., Belgium (Fraeyman et al., 2013). Classes where all products are seen as essentially therapeutically similar include the proton pump inhibitors (PPIs), statins and renin-angiotensin inhibitor drugs, with the latter including angiotensin converting enzyme inhibitors (ACEIs) and angiotensin receptor blockers (ARBs) (Weng et al., 2010; Wettermark et al., 2010; Vončina et al., 2011; Godman et al., 2012a, 2013a; Moon et al., 2010; Martin et al., 2014). As a result, patients can be switched between treatments without compromising care (Usher-Smith et al., 2008; Godman et al., 2011a,b, 2013b; Moon et al., 2010; Martin et al., 2014). Consequently based on available evidence, prescribing selection in these classes should primarily be based on drug acquisition prices as the risks and benefits of different choices should be similar at appropriate doses. As a result, educational activities should be aimed at promoting appropriate doses and improving compliance, which can be poor in patients with chronic diseases (Vončina et al., 2011; Bennie et al., 2012).

Alongside this, instigating a range of measures to address physician and patient concerns with the effectiveness and/or side-effects of generics can also be beneficial. Initiatives include granting substitutability status for generics, publishing lists of substitutable and non-substitutable products, not reimbursing generics where there are concerns with their quality, physician and patient education, encouraging International Non-proprietary Name (INN) prescribing as well as incentivizing pharmacists to talk with patients when substituting or dispensing different branded generics to allay fears and reduce confusion (Godman et al., 2011a, 2012b; Olsson et al., 2014). Such initiatives have resulted in considerable savings as seen in France and more recently in Portugal (Godman et al., 2012a,b).

A number of these strategies are aimed at counter-acting the commercial activities of pharmaceutical companies, who have typically been the principal source of pharmacotherapeutic information among physicians for new and established drugs (Godman et al., 2011a). Published studies suggest marketing costs can be up to one third of a company's income, with companies spending US$53 billion (€40.2) in the US alone in 2004 marketing to physicians alongside lobbying and other indirect strategies (Civaner, 2012; Malmström et al., 2013).

It is recognized that prescribing behavior is complex and that there are different healthcare systems across Europe (Godman et al., 2011a). However, countries need to learn from each other to help maintain the European ideals of equitable and comprehensive healthcare in the face of growing resource pressures. We believe the plethora of different supply- and demand-side measures introduced across Europe to enhance prescribing efficiency will stimulate debates within countries on potential additional initiatives to consider in the future. These can be identified through either single country studies or cross national comparisons of drug utilization and expenditure.

The objectives of this paper are to assess the influence of principally demand-side reforms to enhance prescribing efficiency in ambulatory care before a decision is made to prescribe a particular drug within a class. Subsequently utilize the findings to suggest potential future initiatives that countries could consider to further enhance their prescribing efficiency.

## Methodology

A number of methodologies were used. Firstly, a cross national retrospective observational study of reimbursed utilization and expenditure of the proton pump inhibitors (PPIs) and statins among European countries between 2001 and 2007. These dates were chosen as typically both generic simvastatin and generic omeprazole became available and were reimbursed during this time period among Western European countries (Godman et al., 2011a). Simvastatin was the first major statin to become available as a generic in Europe with generally no or limited utilization of lovastatin. Omeprazole was the first PPI to become available as a generic. Both events resulted in variable demand side initiatives to try and enhance the prescribing of generics first line. In this study, efficiency was broken down into three categories, summarized in Table 1 (Godman et al., 2011a). The three cut-off points for assessing efficiency were validated by health authority and health insurance personnel across Europe (Godman et al., 2011a).

**Table 1:** **Principal measures used to evaluate changes in prescribing efficiency for both the PPIs and statins during the study years as well as categorize countries (Godman et al., 2011a)**.

**Objective**	**Measure**	**Efficiency criteria/comment**
Assessment of overall prescribing efficiency	The increase in utilization rates vs. the increase in reimbursed expenditure over time^*^	3 efficiency criteria:
		No efficiency—rate of increase in expenditure exceeds utilization
		Efficient countries—rate of increase in utilization more than double the rate of increase in expenditure
		Very efficient countries—reimbursed expenditure decreasing over time despite increasing utilization. In the case of statins this also includes considerably increased utilization (over 350% during the study period) with only a limited increase in expenditure (20% or less)
Extent of potential savings from increasing prescribing efficiency	Overall utilization in 2007 (DDD/DID) compared with overall expenditure (€/1000 inhabitants/year), with both measures adjusted for population sizes	Data treated with caution as different co-payment levels for the PPIs and statins in addition to any co-payment for the package

* NB Generic PPIs and statins were often available in Central and Eastern European countries before 2001 influencing the figures. The figures for the Republic of Ireland are distorted by the fact that the GMS population has a greater morbidity than the general population reflected in appreciably higher utilization of pharmaceuticals.

Secondly, single country retrospective observational studies in Scotland (extended from 2007 to 2010), Belgium (1997 to 2009), the Netherlands (2000 to 2010), and the Republic of Srpska (constitutive entity of Bosnia and Herzegovina) (2003 to 2010) to assess reimbursed utilization and expenditure for the PPIs and statins (Bennie et al., 2012; Markovic-Pekovic et al., 2012; van Woerkom et al., 2012; Fraeyman et al., 2013).

Thirdly, a cross national retrospective observational study of reimbursed utilization and expenditure of ACEIs and ARBs among 6 European countries between 2001 and 2007. During this time, multiple measures were introduced by some countries to limit the prescribing of patented ARBs vs. lower cost generic ACEIs (Vončina et al., 2011). The findings were also compared with the influence of prescribing restrictions for the ARBs in the Republic of Srpska (Markovic-Pekovic et al., 2012).

Only administrative databases were used in the various studies to provide accurate data on reimbursed utilization and expenditure (Godman et al., 2011a). As a result, only data on ambulatory care utilization and expenditure data was recorded. This typically includes out-patient but not in-patient data. In all cases, utilization rates were computed using Defined Daily Doses (DDDs) in line with international guidance (WHO, 2003). The latest DDDs were also used, again in line with recommendations (WHO, 2003; Godman et al., 2011a). We did not use Prescribed Daily Doses (PDDs) as there was no access to patient data in the majority of countries studied.

The findings will be supplemented by recently published studies following the availability of generic losartan among the ARBs and generic risperidone among the atypical antipsychotics (Godman et al., 2012b, 2013c,d,e; Martin et al., 2014). The objective is to provide additional guidance to health authorities.

Demand side measures have typically been collated under the “4 Es,” i.e., education, engineering, economics, and enforcement. Examples and definitions include (Wettermark et al., 2009; Godman et al., 2011a):

- **Educational activities**—includes development and distribution of prescribing guidance right through to more intensive strategies including educational outreach visits and benchmarking of physician prescribing habits.
- **Engineering activities**—includes organizational or managerial interventions such as prescribing targets as well as price: volume agreements for single sourced existing products.
- **Economic interventions**—includes devolved budgets with penalties, positive and negative financial incentives for physicians, as well as differential patient co-payments for more expensive products than the current reference molecule.
- **Enforcement**—includes regulations by law such as mandatory generic substitution and compulsory INN prescribing as well as prescribing restrictions.

Reimbursed expenditures were typically captured reflecting the payer perspective of the various studies. The only exceptions were Austria, Germany, and Norway where there are difficulties with disassociating co-payments from total expenditure (Godman et al., 2011a). However, this typically represented only a small proportion of overall expenditure in these three countries. Expenditure data was generally collected in local currency and converted to Euros where pertinent based on the established conversion rates for the country; alternatively an average for the year from national banks (Godman et al., 2011a; Vončina et al., 2011).

There was generally no allowance for inflation in order to directly compare the impact of the different policies over time. In addition, health authorities typically refer to pre-patent loss prices when establishing reimbursed prices for generics (Godman et al., 2010a, 2011a,b; Vončina et al., 2011).

No attempt was made to analyze the appropriateness of prescribing due to the lack of access to patient databases. In addition, the main emphasis of the various studies was on prescribing efficiency once a decision had been made by the physician to prescribe either a PPI, renin-angiotensin inhibitor drug or a statin. As a result, care should not be compromised through encouraging the greater use of generics at appropriate doses (Norman et al., 2008; Usher-Smith et al., 2008; Moon et al., 2010; Godman et al., 2013a; Martin et al., 2014).

## Results

### PPIs and statins 2001 to 2007

**Figure 1:**
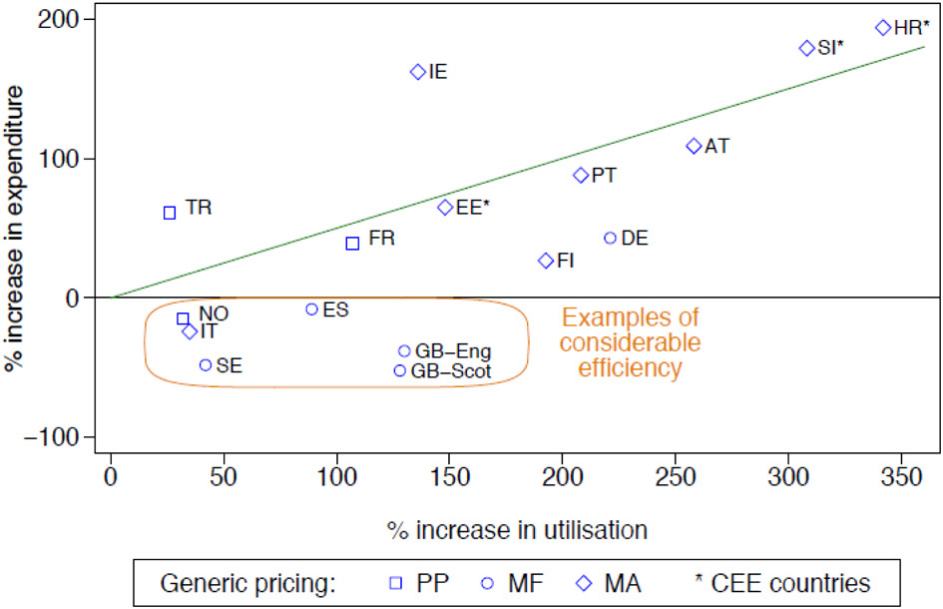
**Rate of increase in expenditure (local currency) vs. the rate of increase in utilisation (DDD based) for the PPIs principally from 2001 to 2007 among European countries (unless stated), with generic pricing approaches divided into three categories (Godman et al., 2011a)**. NB: Generic pricing: PP, Prescriptive pricing; MF, Market Forces; MA, Mixed Approach (Box 1). Standard EU country abbreviations have been used. ES = Catalonia (2007 vs. 2003), EE = 2007 vs. 2004, HR = 2007 vs. 2000, IT = 2008 vs. 2006, NO = 2007 vs. 2004; TR = 2009 vs. 2007.

The influence of the different initiatives in terms of their extent and intensity can be seen in Figure 1 (for the PPIs—similar for the statins) (Godman et al., 2011a). The countries were broken down by:

- Geography—into Central and Eastern European countries and the rest. This is because generic PPIs were generally available earlier in Central and Eastern European countries than Western European countries reducing potential efficiency gains.
- The different approaches to pricing of generics—Prescriptive—PP, Market Forces—MF, Mixed—MA (Box 1) (Godman et al., 2010a).

Box 1 **Different approaches to the pricing of generics in Europe (Godman et al., 2010a).**

**Prescriptive pricing for generics**—this can be either for the molecule (generic and originator) or just for the generic. Health Authorities or Health Insurance Companies mandate price reductions that are necessary for generics to be reimbursed. This is either based on originator prices pre-patent loss, reimbursed prices of generics in other European countries or a mixture of the two.

**Market forces for generics**—In some European countries there is no established price reduction level for the first generic or generics to be reimbursed. This is left to market forces with a number of measures in place to accelerate price reductions.

**Mixture of prescriptive pricing and market forces**—In some European countries price reductions are mandated for the first generic or generics. Market forces after that to further drive down prices.

Countries showing considerable prescribing efficiency, as well as general efficiency, i.e., below the line drawn, are highlighted using the definitions listed in Table 1.

**Figure 2:**
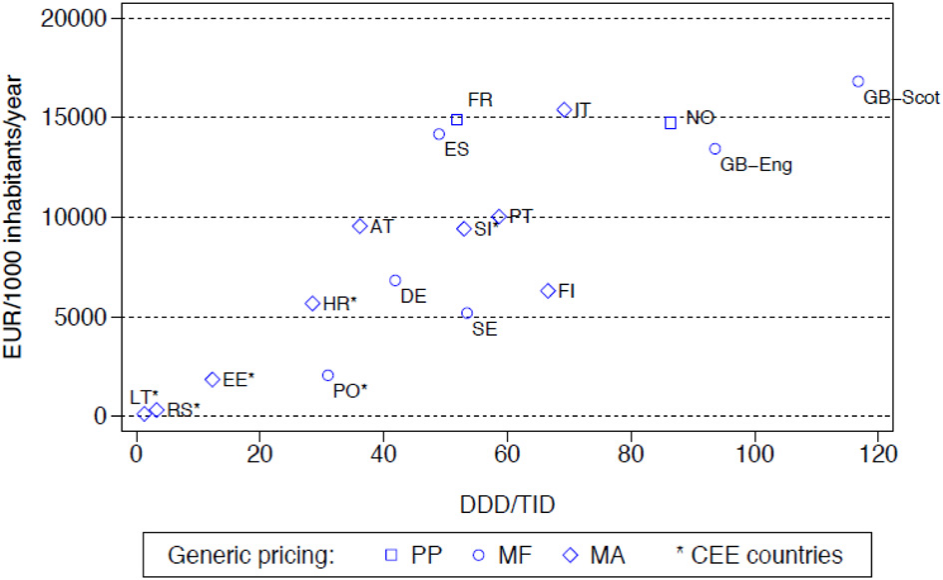
**Utilisation (DDD/TID) and overall expenditure (€/1000 inhabitants/year) for the statins among European countries in 2007 (Italy 2008, Serbia 2008) (Godman et al., 2011a)**. NB: pricing: PP, Prescriptive pricing; MF, Market Forces; MA, Mixed Approach (Box 1). Standard EU country abbreviations have been used. ES = Catalonia.

The differences seen in the rates of prescribing efficiency for the both the **PPIs and statins** between 2001 and 2007 among the various European countries (Figure 1 with similar findings for the statins) were also reflected in considerable differences in overall expenditure in 2007 adjusted for population sizes (Figure 2 with similar findings for the PPIs). The differences in geography and approaches to the pricing of generics have again been highlighted, with overall expenditures affected by whether there are high co-payment levels for the statins (Godman et al., 2011a).

KEY CONCEPT 2. PPIs and statins Multiple and intensive demand-side measures are needed to enhance the prescribing of generic PPIs and statins. Multiple measures resulted in considerable efficiency savings in some European countries with the products in both classes seen as therapeutically similar at appropriate doses in all or nearly all patients. Expenditure/1000 inhabitants/year in Sweden in 2007 was less than one tenth of that seen in the Republic of Ireland, although greater co-morbidity among the patients in Ireland.

Among the Western European countries, expenditure/1000 inhabitants/year in Sweden in 2007 for both the PPIs and statins (Figure 2) with their multiple measures was less than one tenth of that seen in the Republic of Ireland at over €60,000/1000 inhabitants/year (Godman et al., 2010b). However, there was greater morbidity among the selected population in Ireland (Godman et al., 2010b, 2011a).

### PPIs and statins in belgium, the netherlands, the republic of srpska, and scotland

#### Belgium

Multiple reforms including co-payments and reference pricing appreciably enhanced rational prescribing for both the PPIs and statins following generic availability (Fraeyman et al., 2012, 2013). In a recent study, there was an 8-fold increase in PPI utilization between 1997 and 2009 but only 2-fold increase in reimbursed expenditure, helped by decreasing expenditure/DDD for the PPIs from €1.91 in 1997 to €0.52 in 2009. Similarly, a 20-fold increase in statin utilization between 1997 and 2009 but only a 5-fold increase in reimbursed expenditure, helped again by reimbursed expenditure/DDD decreasing from €2.05 in 1997 to €0.57 in 2009 (Fraeyman et al., 2013).

#### The netherlands

Multiple demand-side measures including education, engineering, economics, and enforcement (statins) encouraging the preferential prescribing of generic PPIs and statins resulted in (van Woerkom et al., 2012):

- Reimbursed expenditure for the PPIs fell by 58% in 2010 vs. 2000 despite a 3-fold increase in utilization, helped by increasing utilization of generic omeprazole at only 2% of the pre-patent loss price. The low price for generic omeprazole in the Netherlands was helped by the instigation of a preference pricing policy for multiple sourced products.
- Reimbursed expenditure for the statins fell by 14% in 2010 vs. 2000 despite a 3.8-fold increase in utilization. Again helped by increasing utilization of generic simvastatin at only 2% of the pre-patent loss originator price.

#### The republic of srpska

Multiple reforms have been instigated in the Republic of Srpska over almost a 10-year period. This includes for instance setting the reference price for drugs since 2008 at the lowest INN molecule price (previously set as a median INN price) and reimbursed with a 50% co-payment (previously statins were 30% co-payment and PPIs were not reimbursed at all). At the same time, statins were restricted to specific diseases and conditions precisely defined by ICD-10 codes and not all doses of PPIs were reimbursed. Whilst there was considerably increased utilization of statins at greater than 19-fold between 2004 and 2010, these reforms limited the increase in their reimbursed expenditures to 4-fold during this period. These measures also helped reduce expenditure/DDD for various PPIs between 2008 and 2010 (Markovic-Pekovic et al., 2012).

#### Scotland

Continued multiple demand-side measures in Scotland (education, engineering, and economics) encouraging the preferential prescribing of generic omeprazole and generic statins, coupled with continued measures to lower generic prices through increased transparency, resulted in (Bennie et al., 2012):

- Reimbursed expenditure for the PPIs Scotland in 2010 56% below 2001 levels despite a 3 fold increase in utilization. It was estimated that expenditure on the PPIs in Scotland would have been GB£159 million greater in 2010 for a 5.2 million population assuming similar utilization patterns and costs kept at pre-patent loss prices.
- Reimbursed expenditure for the statins in 2010 in Scotland only 7% above 2001 levels despite a 6.2-fold increase in utilization. Again it was estimated that expenditure for the statins would have been GB£290 million greater in 2010 assuming similar overall utilization patterns and costs kept at the pre-patent loss situation.

### Observational studies assessing demand-side measures among 6 european countries to limit the prescribing of ARBs

The introduction of prescribing restrictions for ARBs in Austria and Croatia restricting the prescribing of ARBs to patients intolerant to ACEIs limited their utilization compared with Portugal (Table 2) where there were only limited demand-side measures combating the marketing activities of pharmaceutical companies (Vončina et al., 2011).

**Table 2:** **% Utilisation of all Angiotensin Converting Enzyme Inhibitors (ACEIs) vs. all renin-angiotensin inhibitor drugs among selected EU countries (DDD basis) from 2001 to 2007 (Vončina et al., 2011)**.

	**2001**	**2002**	**2003**	**2004**	**2005**	**2006**	**2007**
Austria	85	82	81	79	78	76	75
Croatia	98	97	94	91	88	86	87
Portugal	80	75	71	67	64	60	56
Scotland	88	87	85	84	83	82	81

However, the greater intensity of follow-up of prescribing restrictions in Croatia with regular access to patients' histories to check for abuse and potential fines resulted in more limited utilization of ARBs during the study period vs. Austria (Vončina et al., 2011). The introduction of prescribing restrictions for ARBs once reimbursed in the Republic of Srpska, i.e., patients experiencing unwanted side-effects with ACEIs and only upon specialist recommendation with active monitoring by pharmacists, together with 50% co-payment from only selected ARBs and doses otherwise 100% co-payment, also limited their prescribing to just 2% of total renin-angiotensin inhibitor drugs in 2010 (Markovic-Pekovic et al., 2012).

The multiple demand side measures in Scotland (education, engineering, and economics) also limited the prescribing of ARBs compared to Portugal (Table 2) with its limited demand-side measures, with ARB utilization rates similar to Austria and Croatia. This provides guidance to health authorities in countries who are unable to introduce prescribing restrictions. There was also lower utilization of ARBs in Spain (Catalonia) and Sweden compared to Portugal in recent years as a result of their multiple demand-side measures (not shown) (Vončina et al., 2011).

The combination of multiple supply- and demand-side measures helped stabilize expenditure on renin-angiotensin inhibitor drugs in Austria, Croatia, Scotland, Spain (Catalonia), and Sweden between 2001 and 2007 when adjusted for population sizes despite appreciable volume increases, e.g., 159% for Scotland. However, reimbursed expenditure steadily increased in Portugal, 41% higher in 2007 than 2001 (Vončina et al., 2011).

## Discussion

Additional reforms are essential to continue the European ideals given continuing pressures. As such, we consider the findings from the various studies do provide future guidance. This is despite the limitations of observational studies (Godman et al., 2011a; Vončina et al., 2011).

General findings from the first study include more limited utilization, and hence expenditure, of the PPIs and statins among Central and Eastern European countries compared with Western European countries (Figure 2). This is principally due to prescribing restrictions and higher patient co-payments in these countries (Godman et al., 2011a). This endorses the need to document ongoing reforms when comparing utilization rates across countries otherwise there could be concerns with the accuracy of the data (Godman et al., 2011a).

More specific findings include the fact that both supply- and demand-side reforms are essential to maximize prescribing efficiency. They also demonstrate that the influence of demand-side measures appears to be additive, with multiple demand-side measures needed to appreciably affect changes in prescribing patterns and subsequent prescribing efficiency (Godman et al., 2011a). This is illustrated by greater prescribing efficiency in Catalonia (Spain), Sweden and the UK for the PPIs and statins with their multiple and intensive demand-side measures compared with France, Portugal and the Republic of Ireland (Figure 1) with their more limited measures (Godman et al., 2011a). A similar situation is seen with limiting the prescribing of ARBs vs. generic ACEIs in Scotland vs. Portugal (Table 2) (Vončina et al., 2011).

The prescribing of patented ARBs in Europe following the availability of generic losartan followed a similar pattern. Limited demand-side measures in the Republic of Ireland, Scotland, and Spain (Catalonia) resulted in no change in the subsequent utilization of losartan post-generics (Godman et al., 2013c). This was different in Denmark and Sweden with a significant increase in losartan utilization following their various multifaceted demand-side measures including education, engineering, economics, and enforcement (Godman et al., 2013b; Hesse et al., 2013). In one English primary care group, there was no change in the utilization of losartan following generics with no specific demand side measures. However, losartan utilization significantly increased following the instigation of multiple demand-side measures including education, engineering, and economics (Martin et al., 2014). This suggests that there is no “spill over” effect of initiatives between classes to affect changes in prescribing habits even if the classes are closely related (Bennie et al., 2013; Martin et al., 2014). These findings have implications for **future reforms** being considered by health authorities.

KEY CONCEPT 3. Future reforms Both supply- and demand-side measures are essential to improve prescribing efficiency of established medicines. Recent publications suggest that health authorities cannot rely on any “spill over” effect to affect future prescribing changes in similar classes; consequently, multiple initiatives are needed to help health authorities achieve their goals. This includes intensive follow-up of any prescribing restrictions, else health authorities could be disappointed in the outcome.

A similar situation is seen with “enforcement.” Restricting the prescribing of ARBs in Austria, Croatia and the Republic of Srpska and patented statins in Austria and Norway appreciably influenced their utilization (Table 2) (Godman et al., 2011b; Vončina et al., 2011; Markovic-Pekovic et al., 2012). Prescribing restrictions for ARBs introduced in Sweden in 2008 also further reduced their prescribing first line (Wettermark et al., 2010). However, the greater intensity of follow-up of ARB prescribing restrictions in Croatia and the Republic of Srpska, and those for patented statins in Austria, resulted in greater utilization of generics vs. Austria and Norway respectively (Godman et al., 2011b; Vončina et al., 2011; Markovic-Pekovic et al., 2012). This was also seen with the limited impact of prescribing restrictions for esomeprazole in Norway (Godman et al., 2011b). We believe the limited impact observed was due to the fact that specialists in Norway have to verify the diagnosis and recommend therapy before PPIs are reimbursed, and they were not subject to these restrictions (Godman et al., 2011b). In addition, GPs in Norway are reluctant to subsequently change prescriptions. Timing of their introduction may also be important. The prescribing restrictions recently introduced for patented statins in Sweden had limited influence on subsequent utilization patterns (Pettersson et al., 2012). This may be due to the fact that these were introduced some 6 years after intensive regional activities to encourage the preferential prescribing of generic statins (Godman et al., 2011a; Pettersson et al., 2012). Consequently similar to education, engineering, and economics, it is the nature of the follow-up as well as the timing of prescribing restrictions that appears to be important. Failure to appreciate this may result in disappointment for health authorities.

Concentrating on one aspect of reforms, i.e., either supply or demand side measures, but not both, can also reduce potential efficiency gains from the availability of generics. This is illustrated when comparing prescribing efficiency for the statins in Sweden and the UK (England and Scotland) vs. Germany (Figure 2). In Germany in 2007, there was very limited utilization of atorvastatin following the introduction of reference pricing for the class in 2003 at just 2% of overall statin utilization with rosuvastatin not available (Godman et al., 2009). This compares with 21 and 33% respectively on a DDD basis for the appreciably more expensive atorvastatin and rosuvastatin in Sweden and England in 2007 (Godman et al., 2012b). However, comparative expenditures appear similar or greater in Germany compared with Sweden or England due to higher expenditure/DDD for simvastatin (Figure 2). In Korea, just concentrating on cutting pharmaceutical prices without introducing multiple demand-side measures also failed to produce the desired efficiency gains when treating hyperlipidaemia (Kwon et al., 2013).

One aspect of a lower price for generic statins is a lowering of the threshold to prevent coronary vascular disease. Draft guidance in the UK, which is under review, is recommending that statins should now be offered to people who have a 10 per cent or greater 10-year risk of developing CVD (down from 20%) (NICE, 2014).

We are also now seeing countries appearing to learn from each other as resources pressures grow (Godman et al., 2011a). This will continue given continued resource pressures.

However, there are classes where it can be difficult for payers to introduce demand-side measures to appreciably increase the prescribing of generics. This is seen with atypical antipsychotic drugs for treating schizophrenia and bipolar disease where experts as well as national authorities suggest pharmacotherapeutic **treatments should be tailored** to individual patients (Godman et al., 2013d). Recent cross national studies have shown there is a consistent decrease in risperidone utilization as a percentage of all selected atypical antipsychotics (DDD basis) following the availability of generics among a number of European countries (Godman et al., 2013d,e,f). Consequently, health authorities need to wait until more atypical antipsychotic drugs become available as generics before they see significant reductions in expenditure. This is already happening (Godman et al., 2013d).

KEY CONCEPT 4. Tailoring of treatments There are certain classes such as atypical antipsychotic drugs where treatment should be tailored to the individual patient. In these circumstances, it is difficult for health authorities to instigate multiple demand-side measures to affect changes in physician prescribing habits and they need to wait till more drugs become available as generics to realize appreciable savings.

We accept there are limitations with observational studies. However, we believe the consistency of the findings across the various classes will be of interest to health authorities as they plan future supply and demand side measures to further improve their prescribing efficiency. This includes the fact that multiple demand-side measures are needed to affect changes in physician prescribing patterns. Otherwise there is limited change. There also appears to be no “spill over” effect of initiatives between classes even if these are closely related. The timing and follow-up of prescribing restrictions is also important else health authorities could be disappointed with the outcome of such restrictions. Finally, there are some classes where it is difficult to introduce multiple demand-side measures. This includes pharmacotherapy of schizophrenia where treatments should be individualized.

### Conflict of interest statement

The majority of the authors are employed directly by health authorities or health insurance agencies or are advisers to these organisations. No author has any other relevant affiliation or financial involvement with any organisation or entity with a financial interest in or financial conflict with the subject matter or materials discussed in the manuscript.

## References

[B1] Baumgärtel C. Godman B. Malmström R. Andersen M. Abuelkhair M. Abdu S. (2012). What lessons can be learned from the launch of generic clopidogrel? GABI 1, 58–68 10.5639/gabij.2012.0102.016

[B2] Bennie M. Bishop I. Godman B. Campbell S. Miranda J. Finlayson A. E. (2013). Are prescribing initiatives readily transferable across classes: the case of generic losartan in Scotland? Qual. Prim. Care 21, 7–15 23735629

[B3] Bennie M. Godman B. Bishop I. Campbell S. (2012). Multiple initiatives continue to enhance the prescribing efficiency for the proton pump inhibitors and statins in Scotland. Expert Rev. Pharmacoecon. Outcomes Res. 12, 125–130 10.1586/erp.11.98 22280202

[B4] Civaner M. (2012). Sale strategies of pharmaceutical companies in a “pharmerging” country: the problems will not improve if the gaps remain Health Policy 106, 25–32 10.1016/j.healthpol.2012.05.006 22682762

[B5] Dylst P. Vulto A. Godman B. Simoens S. (2013). Generic medicines: solutions for a sustainable drug market? Appl. Health Econ. Health Policy 11, 437–443 10.1007/s40258-013-0043-z 23846572

[B4a] Dylst P. Vulto A. Simoens S. (2011). Tendering for outpatient prescription pharmaceuticals: what can be learned from current practices in Europe? Health Policy 101, 146–152 10.1016/j.healthpol.2011.03.004 21511353

[B6] Experts in chronic myeloid leukemia. (2013). The price of drugs for chronic myeloid leukemia (CML) is a reflection of the unsustainable prices of cancer drugs: from the perspective of a large group of CML experts. Blood 121, 4439–4442 10.1182/blood-2013-03-490003 23620577 PMC4190613

[B7] Ferrario A. Kanavos P. (2013). Managed Entry Agreements for Pharmaceuticals: The European Experience. Available online at: http://ec.europa.eu/enterprise/sectors/healthcare/files/docs/mea_report_en.pdf

[B8] Fraeyman J. Van Hal G. De Loof H. Remmen R. De Meyer G. R. Beutels P. (2012). Potential impact of policy regulation and generic competition on sales of cholesterol lowering medication, antidepressants and acid blocking agents in Belgium. Acta Clin. Belg. 67, 160–171 22897063 10.2143/ACB.67.3.2062650

[B9] Fraeyman J. Van Hal G. Godman B. Beutels P. (2013). The potential influence of various initiatives to improve rational prescribing for proton pump inhibitors and statins in Belgium. Expert Rev. Pharmacoecon. Outcomes Res. 13, 141–151 10.1586/erp.12.88 23402454

[B10] Garattini S. Bertele V. Godman B. Haycox A. Wettermark B. Gustafsson L. L. (2008). Enhancing the rational use of new medicines across European healthcare systems—a position paper. Eur. J. Clin. Pharmacol. 64, 1137–1138 10.1007/s00228-008-0537-z 18688606

[B11] Godman B. Abuelkhair M. Vitry A. Abdu S. Bennie M. Bishop I. (2012a). Payers endorse generics to enhance prescribing efficiency; impact and future implications, a case history approach. GABI 1, 69–83 10.5639/gabij.2012.0102.017

[B12] Godman B. Bennie M. Baumgärtel C. Sovi c-Brkiěić L. Burkhardt T. Fürst J. (2012b). Essential to increase the use of generics in Europe to maintain comprehensive healthcare? Farmeconomia 13 Suppl. 3, 5–20

[B13] Godman B. Bennie M. Bucsics A. Hesse U. Martin A. Miranda J. (2013c). Variable approaches in Europe to the availability of generic losartan; implications for the future. Clin. Ther. 35, e36–e37 10.1016/j.clinthera.2013.07.088

[B14] Godman B. Campbell S. Suh H. S. Finlayson A. E. Bennie M. Gustafsson L. L. (2013a). Ongoing measures to enhance prescribing efficiency across Europe: implications for other countries. J. Health Tech. Assess. 1, 27–42

[B15] Godman B. De Bruyn K. Miranda J. Raschi E. Bennie M. Barbui C. (2013f). Generic atypical antipsychotic drugs in Belgium; their influence and implications. J. Comp. Eff. Res. 2, 551–561 10.2217/cer.13.75 24236794

[B16] Godman B. Persson M. Miranda J. Barbui C. Bennie M. Bennett K. (2013e). Can authorities take full advantage of the availability of generic atypical antipsychotic drugs? Implications for the future. Clin. Ther. 35, e99–e100 10.1016/j.clinthera.2013.07.291

[B17] Godman B. Persson M. Miranda J. Barbui C. Bennie M. Finlayson A. E. (2013d). Can authorities take advantage of the availability of generic atypical antipsychotic drugs? Findings from Sweden and potential implications. J. Pharm. Health Serv. Res. 4, 139–150 10.1111/jphs.12025

[B18] Godman B. Sakshaug S. Berg C. Wettermark B. Haycox A. (2011b). Combination of prescribing restrictions and policies to engineer low prices to reduce reimbursement costs. Expert Rev. Pharmacoecon. Outcomes Res. 11, 121–129 10.1586/erp.10.87 21351864

[B19] Godman B. Schwabe U. Selke G. Wettermark B. (2009). Update of recent reforms in Germany to enhance the quality and efficiency of prescribing of proton pump inhibitors and lipid lowering drugs. Pharmacoeconomics 27, 435–438 10.2165/00019053-200927050-00010 19586083

[B20] Godman B. Shrank W. Andersen M. Berg C. Bishop I. Burkhardt T. (2011a). Policies to enhance prescribing efficiency in Europe: findings and future implications. Front. Pharmacol. 1:141 10.3389/fphar.2010.00141 21833180 PMC3153015

[B21] Godman B. Shrank W. Andersen M. Berg C. Bishop I. Burkhardt T. (2010b). Comparing policies to enhance prescribing efficiency in Europe through increasing generic utilisation: changes seen and global implications. Expert Rev. Pharmacoecon. Outcomes Res. 10, 707–722 10.1586/erp.10.72 21155704

[B22] Godman B. Shrank W. Wettermark B. Andersen M. Bishop I. Burkhardt T. (2010a). Use of generics—a critical cost containment measure for all healthcare professionals in Europe? Pharmaceuticals 3, 2470–2494 10.3390/ph/3082470 PMC4033935 27713363

[B23] Godman B. Wettermark B. Miranda J. Bennie M. Martin A. Malmström R. E. (2013b). Influence of multiple initiatives in Sweden to enhance ARB prescribing efficiency following generic losartan; findings and implications for other countries. Int. J. Clin. Pract. 67, 853–862 10.1111/ijcp.12130 23560825

[B24] Hesse U. Godman B. Petzold M. Martin A. Malmström R. E. (2013). Impact of delisting ARBs, apart from losartan, on ARB utilisation patterns in Denmark; implications for other countries. Appl. Health Econ. Health Pol. 11, 677–685 10.1007/s40258-013-0059-4 24105097

[B25] Klemp M. Frønsdal K. Facey K. HTAi Policy Forum. (2011). What principles should govern the use of managed entry agreements? Int. J. Technol. Assess. Health Care 27, 77–83 10.1017/S0266462310001297 21262072

[B26] Kwon H. Y. Hong J. M. Godman B. Yang B. M. (2013). Price cuts and drug spending in south korea: the case of antihyperlipidemic agents. Health Policy 112, 217–226 10.1016/j.healthpol.2013.08.011 24075008

[B27] Malmström R. E. Godman B. Diogene E. Baumgärtel C. Bennie M. Bishop I. (2013). Dabigatran - a case history demonstrating the need for comprehensive approaches to optimise the use of new drugs. Front. Pharmacol. 4, 1–19 10.3389/fphar.2013.00039 23717279 PMC3653065

[B28] Markovic-Pekovic V. Ranko Škrbić R. Godman B. Gustafsson L. L. (2012). Ongoing initiatives in the Republic of Srpska to enhance prescribing efficiency: influence and future directions. Expert Rev. Pharmacoecon. Outcomes Res. 5, 661–671 10.1586/erp.12.48 23186404

[B29] Martin A. Godman B. Miranda J. Tilstone J. Saleem N. Olsson E. (2014). Measures to improve angiotensin receptor blocker prescribing efficiency in the UK: findings and implications. J. Comp. Eff. Res. 3, 41–51 10.2217/cer.13.83 24345256

[B30] Moon J. Flett A. Godman B. Grosso A. M. Wierzbicki A. S. (2010). Getting better value from the NHS drug budget. BMJ 341:c6449 10.1136/bmj.c6449 21169320

[B31] NICE. (2014). Thousands More Could be Offered Statins. Available online at: http://www.nice.org.uk/newsroom/news/ThousandsMoreCouldBeOfferedStatins.jsp (Accessed March 2014).

[B32] Norman C. Zarrinkoub R. Hasselström J. Godman B. Granath F. Wettermark B. (2008). Potential savings without compromising the quality of care. Int. J. Clin. Pract. 63, 1320–1326 10.1111/j.1742-1241.2009.02129.x 19691615

[B33] Olsson E. Ingman P. Ahmed A. Sporrong S. (2014). Pharmacist-patient communication in Swedish community pharmacists. Res. Social Adm. Pharm. 10, 149–155 10.1016/j.sapharm.2013.03.001 23591412

[B34] Pettersson B. Hoffmann M. Per Wändell P. Levin L. Å. (2012). Utilization and costs of lipid modifying therapies following health technology assessment for the new reimbursement scheme in Sweden. Health Policy 104, 84–91 10.1016/j.healthpol.2011.10.010 22115548

[B35] Usher-Smith J. Ramsbottom T. Pearmain H. Kirby M. (2008). Evaluation of the clinical outcomes of switching patients from atorvastatin to simvastatin and losartan to candesartan in a primary care setting: 2 years on. Int. J. Clin. Pract. 62, 480–484 10.1111/j.1742-1241.2007.01690.x 18201178

[B36] van Woerkom M. Piepenbrink J. F. Godman B. Metz J. D. Campbell S. Bennie M. (2012). Ongoing measures to enhance the efficiency of prescribing of PPIs and statins in the Netherlands; influence and future implications. J. Comp. Eff. Res. 1, 527–538 10.2217/cer.12.52 24236472

[B37] Vončina L. Strizrep T. Godman B. Bennie M. Bishop I. Campbell S. (2011). Influence of demand-side measures to enhance renin–angiotensin prescribing efficiency in Europe: implications for the future. Expert Rev. Pharmacoecon. Outcomes Res. 11, 469–479 10.1586/erp.11.42 21831028

[B38] Weng T. C. Yang Y. H. Lin S. J. Tai S. H. (2010). A systematic review and meta-analysis on the therapeutic equivalence of statins. J. Clin. Pharm. Ther. 35, 139–151 10.1111/j.1365-2710.2009.01085.x 20456733

[B39] Wettermark B. Godman B. Jacobsson B. Haaijer-Ruskamp F. (2009). Soft regulations in pharmaceutical policymaking—an overview of current approaches and their consequences. Appl. Health Econ. Health Policy 7, 1–11 10.1007/BF03256147 19799468

[B40] Wettermark B. Godman B. Neovius M. Hedberg N. Mellgren T. O. Kahan T. (2010). Initial effects of a reimbursement restriction to improve the cost-effectiveness of antihypertensive treatment. Health Policy 94, 221–229 10.1016/j.healthpol.2009.09.014 19879009

[B41] World Health Organization (WHO). (2003). Introduction to Drug Utilisation Research. Available online at: http://www.who.int/medicines/areas/quality_safety/safety_efficacy/Drug%20utilization%20research.pdf

